# Supplementary material for: Pyruvate Dehydrogenase Kinase 1 inhibition mediated oxidative phosphorylation enhancement in cartilage promotes osteoarthritis progression
Source: BMC Musculoskelet Disord. 2023 Jul 20;24:597. doi: 10.1186/s12891-023-06585-6 (PMC10357736; doi:10.1186/s12891-023-06585-6)
Supplement: Supplementary file 1 — Additional file 1. [file 12891_2023_6585_MOESM1_ESM.zip › BP.Gsea.1653623667859/GOBP_CELLULAR_RESPONSE_TO_INTERLEUKIN_1.html]

Details for gene set GOBP\_CELLULAR\_RESPONSE\_TO\_INTERLEUKIN\_1[GSEA]

|  || Dataset | 55457\_collapsed\_to\_symbols.cls.cls #OA\_versus\_normal.cls.cls #OA\_versus\_normal\_repos |
| Phenotype | cls.cls#OA\_versus\_normal\_repos |
| Upregulated in class | normal |
| GeneSet | GOBP\_CELLULAR\_RESPONSE\_TO\_INTERLEUKIN\_1 |
| Enrichment Score (ES) | -0.40295288 |
| Normalized Enrichment Score (NES) | -1.7371294 |
| Nominal p-value | 0.0 |
| FDR q-value | 0.11932628 |
| FWER p-Value | 1.0 |
Table: GSEA Results Summary

  

Fig 1: Enrichment plot: GOBP\_CELLULAR\_RESPONSE\_TO\_INTERLEUKIN\_1      
 Profile of the Running ES Score & Positions of GeneSet Members on the Rank Ordered List

  

| SYMBOL | TITLE | RANK IN GENE LIST | RANK METRIC SCORE | RUNNING ES | CORE ENRICHMENT || 1 | TANK | TRAF family member associated NFKB activator [Source:HGNC Symbol;Acc:HGNC:11562] | 439 | 0.690 | -0.0126 | No |
| 2 | KMO | kynurenine 3-monooxygenase [Source:HGNC Symbol;Acc:HGNC:6381] | 694 | 0.595 | -0.0138 | No |
| 3 | MAPK3 | mitogen-activated protein kinase 3 [Source:HGNC Symbol;Acc:HGNC:6877] | 757 | 0.579 | -0.0005 | No |
| 4 | HYAL1 | hyaluronidase 1 [Source:HGNC Symbol;Acc:HGNC:5320] | 997 | 0.518 | -0.0029 | No |
| 5 | PYCARD | PYD and CARD domain containing [Source:HGNC Symbol;Acc:HGNC:16608] | 1164 | 0.484 | -0.0007 | No |
| 6 | CD47 | CD47 molecule [Source:HGNC Symbol;Acc:HGNC:1682] | 1282 | 0.461 | 0.0047 | No |
| 7 | ZNF675 | zinc finger protein 675 [Source:HGNC Symbol;Acc:HGNC:30768] | 1489 | 0.431 | 0.0021 | No |
| 8 | MAP3K7 | mitogen-activated protein kinase kinase kinase 7 [Source:HGNC Symbol;Acc:HGNC:6859] | 1701 | 0.399 | -0.0019 | No |
| 9 | CCL19 | C-C motif chemokine ligand 19 [Source:HGNC Symbol;Acc:HGNC:10617] | 2060 | 0.355 | -0.0187 | No |
| 10 | SOX9 | SRY-box transcription factor 9 [Source:HGNC Symbol;Acc:HGNC:11204] | 2089 | 0.351 | -0.0099 | No |
| 11 | SFRP1 | secreted frizzled related protein 1 [Source:HGNC Symbol;Acc:HGNC:10776] | 2503 | 0.303 | -0.0326 | No |
| 12 | MAPK13 | mitogen-activated protein kinase 13 [Source:HGNC Symbol;Acc:HGNC:6875] | 2544 | 0.298 | -0.0264 | No |
| 13 | PTGIS | prostaglandin I2 synthase [Source:HGNC Symbol;Acc:HGNC:9603] | 2654 | 0.284 | -0.0260 | No |
| 14 | AQP4 | aquaporin 4 [Source:HGNC Symbol;Acc:HGNC:637] | 2923 | 0.258 | -0.0388 | No |
| 15 | CCL13 | C-C motif chemokine ligand 13 [Source:HGNC Symbol;Acc:HGNC:10611] | 3379 | 0.214 | -0.0676 | No |
| 16 | ST18 | ST18 C2H2C-type zinc finger transcription factor [Source:HGNC Symbol;Acc:HGNC:18695] | 3560 | 0.197 | -0.0755 | No |
| 17 | SIRPA | signal regulatory protein alpha [Source:HGNC Symbol;Acc:HGNC:9662] | 3599 | 0.193 | -0.0724 | No |
| 18 | RPS6KA4 | ribosomal protein S6 kinase A4 [Source:HGNC Symbol;Acc:HGNC:10433] | 3702 | 0.185 | -0.0745 | No |
| 19 | FGB | fibrinogen beta chain [Source:HGNC Symbol;Acc:HGNC:3662] | 3721 | 0.183 | -0.0702 | No |
| 20 | PLCB1 | phospholipase C beta 1 [Source:HGNC Symbol;Acc:HGNC:15917] | 3724 | 0.183 | -0.0646 | No |
| 21 | XCL1 | X-C motif chemokine ligand 1 [Source:HGNC Symbol;Acc:HGNC:10645] | 3789 | 0.178 | -0.0640 | No |
| 22 | RELA | "RELA proto-oncogene, NF-kB subunit [Source:HGNC Symbol;Acc:HGNC:9955]" | 3942 | 0.161 | -0.0708 | No |
| 23 | UPF1 | UPF1 RNA helicase and ATPase [Source:HGNC Symbol;Acc:HGNC:9962] | 4434 | 0.118 | -0.1054 | No |
| 24 | NFKB1 | nuclear factor kappa B subunit 1 [Source:HGNC Symbol;Acc:HGNC:7794] | 4626 | 0.102 | -0.1172 | No |
| 25 | CCL18 | C-C motif chemokine ligand 18 [Source:HGNC Symbol;Acc:HGNC:10616] | 4659 | 0.099 | -0.1165 | No |
| 26 | VRK2 | VRK serine/threonine kinase 2 [Source:HGNC Symbol;Acc:HGNC:12719] | 4918 | 0.078 | -0.1342 | No |
| 27 | MYLK3 | myosin light chain kinase 3 [Source:HGNC Symbol;Acc:HGNC:29826] | 5294 | 0.048 | -0.1620 | No |
| 28 | IL17A | interleukin 17A [Source:HGNC Symbol;Acc:HGNC:5981] | 5758 | 0.011 | -0.1978 | No |
| 29 | TRAF6 | TNF receptor associated factor 6 [Source:HGNC Symbol;Acc:HGNC:12036] | 5761 | 0.011 | -0.1976 | No |
| 30 | CX3CL1 | C-X3-C motif chemokine ligand 1 [Source:HGNC Symbol;Acc:HGNC:10647] | 5764 | 0.011 | -0.1974 | No |
| 31 | IKBKB | inhibitor of nuclear factor kappa B kinase subunit beta [Source:HGNC Symbol;Acc:HGNC:5960] | 5914 | 0.001 | -0.2090 | No |
| 32 | FGG | fibrinogen gamma chain [Source:HGNC Symbol;Acc:HGNC:3694] | 5997 | -0.004 | -0.2153 | No |
| 33 | ANKRD1 | ankyrin repeat domain 1 [Source:HGNC Symbol;Acc:HGNC:15819] | 6416 | -0.037 | -0.2468 | No |
| 34 | TOLLIP | toll interacting protein [Source:HGNC Symbol;Acc:HGNC:16476] | 6438 | -0.038 | -0.2472 | No |
| 35 | CCL1 | C-C motif chemokine ligand 1 [Source:HGNC Symbol;Acc:HGNC:10609] | 6462 | -0.039 | -0.2478 | No |
| 36 | GBP1 | guanylate binding protein 1 [Source:HGNC Symbol;Acc:HGNC:4182] | 6490 | -0.042 | -0.2486 | No |
| 37 | CCL5 | C-C motif chemokine ligand 5 [Source:HGNC Symbol;Acc:HGNC:10632] | 6584 | -0.048 | -0.2543 | No |
| 38 | MYD88 | MYD88 innate immune signal transduction adaptor [Source:HGNC Symbol;Acc:HGNC:7562] | 6638 | -0.052 | -0.2568 | No |
| 39 | CD40 | CD40 molecule [Source:HGNC Symbol;Acc:HGNC:11919] | 6825 | -0.065 | -0.2693 | No |
| 40 | CCL21 | C-C motif chemokine ligand 21 [Source:HGNC Symbol;Acc:HGNC:10620] | 6856 | -0.068 | -0.2696 | No |
| 41 | CCL11 | C-C motif chemokine ligand 11 [Source:HGNC Symbol;Acc:HGNC:10610] | 6907 | -0.073 | -0.2712 | No |
| 42 | NR1D1 | nuclear receptor subfamily 1 group D member 1 [Source:HGNC Symbol;Acc:HGNC:7962] | 6974 | -0.078 | -0.2739 | No |
| 43 | MMP2 | matrix metallopeptidase 2 [Source:HGNC Symbol;Acc:HGNC:7166] | 7037 | -0.082 | -0.2761 | No |
| 44 | GBP2 | guanylate binding protein 2 [Source:HGNC Symbol;Acc:HGNC:4183] | 7335 | -0.105 | -0.2960 | No |
| 45 | TNIP2 | TNFAIP3 interacting protein 2 [Source:HGNC Symbol;Acc:HGNC:19118] | 7659 | -0.131 | -0.3171 | No |
| 46 | CCL14 | C-C motif chemokine ligand 14 [Source:HGNC Symbol;Acc:HGNC:10612] | 8239 | -0.174 | -0.3569 | No |
| 47 | CCL7 | C-C motif chemokine ligand 7 [Source:HGNC Symbol;Acc:HGNC:10634] | 8351 | -0.182 | -0.3598 | No |
| 48 | AKAP12 | A-kinase anchoring protein 12 [Source:HGNC Symbol;Acc:HGNC:370] | 8414 | -0.186 | -0.3588 | No |
| 49 | CCL24 | C-C motif chemokine ligand 24 [Source:HGNC Symbol;Acc:HGNC:10623] | 8833 | -0.220 | -0.3846 | No |
| 50 | RBMX | RNA binding motif protein X-linked [Source:HGNC Symbol;Acc:HGNC:9910] | 8938 | -0.227 | -0.3856 | No |
| 51 | ZC3H12A | zinc finger CCCH-type containing 12A [Source:HGNC Symbol;Acc:HGNC:26259] | 9030 | -0.234 | -0.3853 | No |
| 52 | PCK1 | phosphoenolpyruvate carboxykinase 1 [Source:HGNC Symbol;Acc:HGNC:8724] | 9100 | -0.239 | -0.3832 | No |
| 53 | CCL15 | C-C motif chemokine ligand 15 [Source:HGNC Symbol;Acc:HGNC:10613] | 9354 | -0.260 | -0.3948 | Yes |
| 54 | ADAMTS12 | ADAM metallopeptidase with thrombospondin type 1 motif 12 [Source:HGNC Symbol;Acc:HGNC:14605] | 9360 | -0.260 | -0.3870 | Yes |
| 55 | IRAK1 | interleukin 1 receptor associated kinase 1 [Source:HGNC Symbol;Acc:HGNC:6112] | 9377 | -0.262 | -0.3801 | Yes |
| 56 | RPS6KA5 | ribosomal protein S6 kinase A5 [Source:HGNC Symbol;Acc:HGNC:10434] | 9406 | -0.265 | -0.3739 | Yes |
| 57 | CAMP | cathelicidin antimicrobial peptide [Source:HGNC Symbol;Acc:HGNC:1472] | 9471 | -0.271 | -0.3704 | Yes |
| 58 | CCL22 | C-C motif chemokine ligand 22 [Source:HGNC Symbol;Acc:HGNC:10621] | 9496 | -0.274 | -0.3637 | Yes |
| 59 | CCL23 | C-C motif chemokine ligand 23 [Source:HGNC Symbol;Acc:HGNC:10622] | 9610 | -0.284 | -0.3636 | Yes |
| 60 | CCL2 | C-C motif chemokine ligand 2 [Source:HGNC Symbol;Acc:HGNC:10618] | 9819 | -0.303 | -0.3704 | Yes |
| 61 | CCL17 | C-C motif chemokine ligand 17 [Source:HGNC Symbol;Acc:HGNC:10615] | 9893 | -0.311 | -0.3663 | Yes |
| 62 | USP10 | ubiquitin specific peptidase 10 [Source:HGNC Symbol;Acc:HGNC:12608] | 9955 | -0.317 | -0.3612 | Yes |
| 63 | ZBP1 | Z-DNA binding protein 1 [Source:HGNC Symbol;Acc:HGNC:16176] | 10164 | -0.337 | -0.3668 | Yes |
| 64 | IL1R2 | interleukin 1 receptor type 2 [Source:HGNC Symbol;Acc:HGNC:5994] | 10262 | -0.347 | -0.3635 | Yes |
| 65 | HYAL2 | hyaluronidase 2 [Source:HGNC Symbol;Acc:HGNC:5321] | 10420 | -0.363 | -0.3644 | Yes |
| 66 | CCL8 | C-C motif chemokine ligand 8 [Source:HGNC Symbol;Acc:HGNC:10635] | 10529 | -0.376 | -0.3610 | Yes |
| 67 | CCL20 | C-C motif chemokine ligand 20 [Source:HGNC Symbol;Acc:HGNC:10619] | 10859 | -0.411 | -0.3738 | Yes |
| 68 | IRAK4 | interleukin 1 receptor associated kinase 4 [Source:HGNC Symbol;Acc:HGNC:17967] | 10874 | -0.413 | -0.3619 | Yes |
| 69 | HAS2 | hyaluronan synthase 2 [Source:HGNC Symbol;Acc:HGNC:4819] | 10980 | -0.428 | -0.3567 | Yes |
| 70 | IL1RN | interleukin 1 receptor antagonist [Source:HGNC Symbol;Acc:HGNC:6000] | 11014 | -0.432 | -0.3457 | Yes |
| 71 | OTUD4 | OTU deubiquitinase 4 [Source:HGNC Symbol;Acc:HGNC:24949] | 11055 | -0.438 | -0.3351 | Yes |
| 72 | CCL16 | C-C motif chemokine ligand 16 [Source:HGNC Symbol;Acc:HGNC:10614] | 11140 | -0.449 | -0.3276 | Yes |
| 73 | CCL4 | C-C motif chemokine ligand 4 [Source:HGNC Symbol;Acc:HGNC:10630] | 11211 | -0.458 | -0.3187 | Yes |
| 74 | EDN1 | endothelin 1 [Source:HGNC Symbol;Acc:HGNC:3176] | 11276 | -0.468 | -0.3090 | Yes |
| 75 | NKX3-1 | NK3 homeobox 1 [Source:HGNC Symbol;Acc:HGNC:7838] | 11348 | -0.478 | -0.2995 | Yes |
| 76 | KLF2 | Kruppel like factor 2 [Source:HGNC Symbol;Acc:HGNC:6347] | 11350 | -0.478 | -0.2846 | Yes |
| 77 | CCL3 | C-C motif chemokine ligand 3 [Source:HGNC Symbol;Acc:HGNC:10627] | 11465 | -0.497 | -0.2779 | Yes |
| 78 | MAPK11 | mitogen-activated protein kinase 11 [Source:HGNC Symbol;Acc:HGNC:6873] | 11501 | -0.501 | -0.2649 | Yes |
| 79 | HIF1A | hypoxia inducible factor 1 subunit alpha [Source:HGNC Symbol;Acc:HGNC:4910] | 11833 | -0.556 | -0.2733 | Yes |
| 80 | EGR1 | early growth response 1 [Source:HGNC Symbol;Acc:HGNC:3238] | 12052 | -0.607 | -0.2713 | Yes |
| 81 | IL1B | interleukin 1 beta [Source:HGNC Symbol;Acc:HGNC:5992] | 12105 | -0.622 | -0.2558 | Yes |
| 82 | YY1 | YY1 transcription factor [Source:HGNC Symbol;Acc:HGNC:12856] | 12229 | -0.653 | -0.2450 | Yes |
| 83 | CACTIN | "cactin, spliceosome C complex subunit [Source:HGNC Symbol;Acc:HGNC:29938]" | 12270 | -0.668 | -0.2271 | Yes |
| 84 | CXCL8 | C-X-C motif chemokine ligand 8 [Source:HGNC Symbol;Acc:HGNC:6025] | 12297 | -0.676 | -0.2079 | Yes |
| 85 | IRAK3 | interleukin 1 receptor associated kinase 3 [Source:HGNC Symbol;Acc:HGNC:17020] | 12383 | -0.703 | -0.1925 | Yes |
| 86 | IL1R1 | interleukin 1 receptor type 1 [Source:HGNC Symbol;Acc:HGNC:5993] | 12419 | -0.713 | -0.1729 | Yes |
| 87 | IL1RL2 | interleukin 1 receptor like 2 [Source:HGNC Symbol;Acc:HGNC:5999] | 12435 | -0.719 | -0.1515 | Yes |
| 88 | IL6 | interleukin 6 [Source:HGNC Symbol;Acc:HGNC:6018] | 12460 | -0.730 | -0.1304 | Yes |
| 89 | ADAMTS7 | ADAM metallopeptidase with thrombospondin type 1 motif 7 [Source:HGNC Symbol;Acc:HGNC:223] | 12466 | -0.733 | -0.1078 | Yes |
| 90 | HES1 | hes family bHLH transcription factor 1 [Source:HGNC Symbol;Acc:HGNC:5192] | 12676 | -0.849 | -0.0975 | Yes |
| 91 | INHBB | inhibin subunit beta B [Source:HGNC Symbol;Acc:HGNC:6067] | 12680 | -0.851 | -0.0711 | Yes |
| 92 | RORA | RAR related orphan receptor A [Source:HGNC Symbol;Acc:HGNC:10258] | 12696 | -0.862 | -0.0452 | Yes |
| 93 | MYC | "MYC proto-oncogene, bHLH transcription factor [Source:HGNC Symbol;Acc:HGNC:7553]" | 12801 | -0.970 | -0.0229 | Yes |
| 94 | CEBPB | CCAAT enhancer binding protein beta [Source:HGNC Symbol;Acc:HGNC:1834] | 12804 | -0.973 | 0.0075 | Yes |
Table: GSEA details [plain text format]

  

Fig 2: GOBP\_CELLULAR\_RESPONSE\_TO\_INTERLEUKIN\_1      
 Blue-Pink O' Gram in the Space of the Analyzed GeneSet

  

Fig 3: GOBP\_CELLULAR\_RESPONSE\_TO\_INTERLEUKIN\_1: Random ES distribution      
 Gene set null distribution of ES for **GOBP\_CELLULAR\_RESPONSE\_TO\_INTERLEUKIN\_1**
